# Genome-wide crosstalk between steroid receptors in breast and prostate cancers

**DOI:** 10.1530/ERC-21-0038

**Published:** 2021-06-16

**Authors:** Ville Paakinaho, Jorma J Palvimo

**Affiliations:** 1 Institute of Biomedicine, School of Medicine, University of Eastern Finland, Kuopio, Finland

**Keywords:** androgen receptor, estrogen receptor, glucocorticoid receptor, progesterone receptor, breast cancer, prostate cancer, chromatin, crosstalk

## Abstract

Steroid receptors (SRs) constitute an important class of signal-dependent transcription factors (TFs). They regulate a variety of key biological processes and are crucial drug targets in many disease states. In particular, estrogen (ER) and androgen receptors (AR) drive the development and progression of breast and prostate cancer, respectively. Thus, they represent the main specific drug targets in these diseases. Recent evidence has suggested that the crosstalk between signal-dependent TFs is an important step in the reprogramming of chromatin sites; a signal-activated TF can expand or restrict the chromatin binding of another TF. This crosstalk can rewire gene programs and thus alter biological processes and influence the progression of disease. Lately, it has been postulated that there may be an important crosstalk between the AR and the ER with other SRs. Especially, progesterone (PR) and glucocorticoid receptor (GR) can reprogram chromatin binding of ER and gene programs in breast cancer cells. Furthermore, GR can take the place of AR in antiandrogen-resistant prostate cancer cells. Here, we review the current knowledge of the crosstalk between SRs in breast and prostate cancers. We emphasize how the activity of ER and AR on chromatin can be modulated by other SRs on a genome-wide scale. We also highlight the knowledge gaps in the interplay of SRs and their complex interactions with other signaling pathways and suggest how to experimentally fill in these gaps.

## Introduction

The nuclear receptor (NR) superfamily consists of 48 transcription factors (TFs), most, if not all, are key regulators of essential biological functions, such as development, metabolism, and reproduction. Notably, many of the NRs are associated with multiple disease states and serious illnesses (Mangelsdorf *et al.* 1995, Achermann *et al.* 2017, Lazar 2017). The majority of the NRs have the same domain structure with a variable N-terminal domain (NTD), a DNA-binding domain (DBD), a hinge region, and a C-terminal ligand/hormone-binding domain (LBD). Transcriptional coregulators often interact selectively with the LBD, depending on the conformational change which has been induced by different ligands (Perissi & Rosenfeld 2005, Arnal *et al.* 2017). The DBD is the best conserved domain of the NRs, which distinguishes these receptors from other TFs (Lambert *et al.* 2018). NRs are associated with several human cancers. Depending on NRs’ cellular context and composition, they can function either as oncogenes or tumor suppressors (Holbeck *et al.* 2010, Dhiman *et al.* 2018). A subfamily of the NRs, steroid receptors (SRs) are particularly associated with breast cancer (BCa) and prostate cancer (PCa); these are cancers whose development and growth are initially steroid hormone-dependent (Metcalfe *et al.* 2018, Dhiman *et al.* 2018).

The family of SRs consists of estrogen receptor (ER), and 3-ketosteroid receptors (NR3Cs), glucocorticoid (GR, NR3C1), mineralocorticoid (MR, NR3C2), progesterone (PR, NR3C3), and androgen (AR, NR3C4) receptor (Carson-Jurica *et al.* 1990). ERs are encoded by two different genes, ERα (NR3A1) and ERβ (NR3A2) (Arnal *et al.* 2017). Unless specifically indicated, we will use the abbreviation ER to represent ERα from now on. In the absence of a hormonal stimulus, the SRs usually, but not invariably, reside in the cytoplasm associated with a chaperone complex which maintains the SR in an inactive form but still capable of binding a hormonal ligand (Arnal *et al.* 2017, Timmermans *et al.* 2019). Subsequently, after ligand binding, a conformational change occurs in the SR, resulting in the dissociation of the chaperone complex, its translocation to the nucleus, oligomerization, and binding to regulatory elements at enhancers on chromatin. The SRs have been widely assumed to form homodimers, but recent evidence suggests that the SRs can form higher oligomerization states, such as tetramers (Presman *et al.* 2016, Fuentes-Prior *et al.* 2019). On chromatin, SRs bind often, but not always, to hormone response elements (HREs) that usually consist of an imperfect palindrome sequence of two 6 bp half-sites separated by three nucleotides. The response element for ER (ERE) is commonly 5’-AGGTCAnnnTGACCT-3’, while the corresponding response element for the 3-ketosteroid receptors (GRE, MRE, PRE, ARE) is 5’-GGTACAnnnTGTTCT-3’ (Coons *et al.* 2017). The binding sites of SRs contain a variable number of response element sequences; these sites mostly occur distal to gene promoters (Everett & Lazar 2013). At these sites, SRs recruit a variety of other TFs and transcriptional coregulators, coactivators and corepressors, to chromatin, ultimately influencing the transcription and expression of their target genes (Perissi & Rosenfeld 2005, Lempiäinen *et al.* 2017, Papachristou *et al.* 2018). Most of the coregulators recruited by the SRs harbor histone-modifying and chromatin-remodeling activities and they are often shared between the SRs (Lempiäinen *et al.* 2017). Since corepressors are expressed at a large excess over coactivators (Gillespie *et al.* 2020), competition between coactivators, for example, through squelching, could occur when multiple SRs are activated. Finally, since in most cases, the SR-binding sites are located outside of the target gene promoters, the SRs are thought to enable the formation of chromatin loops with the promoters (Fullwood *et al.* 2009, D’Ippolito *et al.* 2018, Zhang *et al.* 2019).

In hormone-dependent cancers, such as BCa and PCa, ER’s growth-promoting transcriptional programs as well as those of AR are considered as the key drivers of cancer development and progression (Swinstead *et al.* 2018, Feng & He 2019). Thus, in the therapy of these cancers, the ER and the AR are targeted by antagonist compounds (Metcalfe *et al.* 2018). In addition, coregulators recruited by the ER and AR have recently emerged as potential targets for cancer therapies (Groner & Brown 2017, Wimalasena *et al.* 2020). The druggable coregulators include EP300 and various proteins of the bromodomain and extra-terminal (BET) family (Lasko *et al.* 2017, Murakami *et al.* 2019, Gilan *et al.* 2020). The concomitant targeting of the ER or the AR with a coregulator could help to resolve drug resistance occurring with single drug treatments (Metcalfe *et al.* 2018, Boumahdi & de Sauvage 2020, Carceles-Cordon *et al.* 2020). Interestingly, also other SRs have emerged as important 'coregulators' for the ER and AR in cancer cells (Kach *et al.* 2015). Since recent work has shown that TFs can modulate chromatin binding and the activity of other TFs through multiple mechanisms, including cooperative binding, tethering and assisted loading (Long *et al.* 2016, Morgunova & Taipale 2017, Swinstead *et al.* 2018), here we reviewed the crosstalk and interplay between SRs from the view of steroid-dependency in BCa and PCa. We will focus on how signaling via ER and AR in BCa and PCa, respectively, can be altered by other SRs on a genome-wide scale.

## Breast cancer

BCa is the most common cancer in women and among the leading causes of cancer deaths in both the United States and Finland (Centers for Disease Control and Prevention 2017, Finnish Cancer Registry 2018). Estrogens, principally estradiol (E2), and ER are considered as the main drivers of BCa development and progression (Swinstead *et al.* 2018). BCa is primarily classified by the expression of ER, PR and human EGF receptor 2 (HER2/ERBB2), and divided into three main subtypes ER+/PR+/HER2+, ER-/PR-/HER2+ and ER-/PR-/HER2- (Waks & Winer 2019). The latter subtype is commonly indicated as triple negative BCa (TNBC), that is, the cancer cells do not express any of the three proteins. ER+ BCa is usually treated with ER antagonists, such as tamoxifen or fulvestrant, that compete with E2 for binding to the LBD (Arnal *et al.* 2017, Waks & Winer 2019). In addition, aromatase inhibitors (AI) can be used to block the synthesis of E2. However, resistance to AI or ER antagonist treatment can occur. The mechanisms underlying the resistance vary, including ER mutations generating ligand-independent receptor forms (Hanker *et al.* 2020). The TNBC is usually treated with chemotherapy; however, as highlighted in the next sections, in addition to ER and PR, other SRs could be considered as potential and alternative targets of therapy. Interestingly, in ER+ BCa cells, the genome-wide binding of GR to chromatin is similar to that of ER, while AR binds in a similar manner as PR (Kittler *et al.* 2013). Furthermore, in male and female BCa patients, AR, PR, and GR have been shown to occupy ER-binding chromatin sites (Severson *et al.* 2018). Thus, it is important to understand how these SRs interact on chromatin and regulate transcription and consequently how they influence the development and progression of BCa. We will focus on the data derived from genome-wide experiments.

### Genome-wide crosstalk between the ER and the PR in BCa

Historically, PR expression in BCa has been used as a proxy for the function of ER in the disease. *PGR* encoding PR is a well-known E2-regulated gene, and its expression is thought to reflect the transcriptional activity of ER (Creighton *et al.* 2009, Siersbæk *et al.* 2018). Thus, both receptors are expressed at a similar frequency of ~50–80% in all BCa cases although the PR is not expressed in all ER+BCa patients, the actual percentage is around 75% (McGuire *et al.* 1978, Swinstead *et al.* 2018). In addition to as acting as TFs, both ER and PR have been shown to function as local and global genome organizers in their unliganded state in BCa cells (Le Dily *et al*. 2019). This suggests that the expression status of the ER and PR could also influence BCa survival through the regulation of chromosome organization. Indeed, the organization often changes during the development and progression of cancer (See *et al.* 2019).

**Figure 1:**
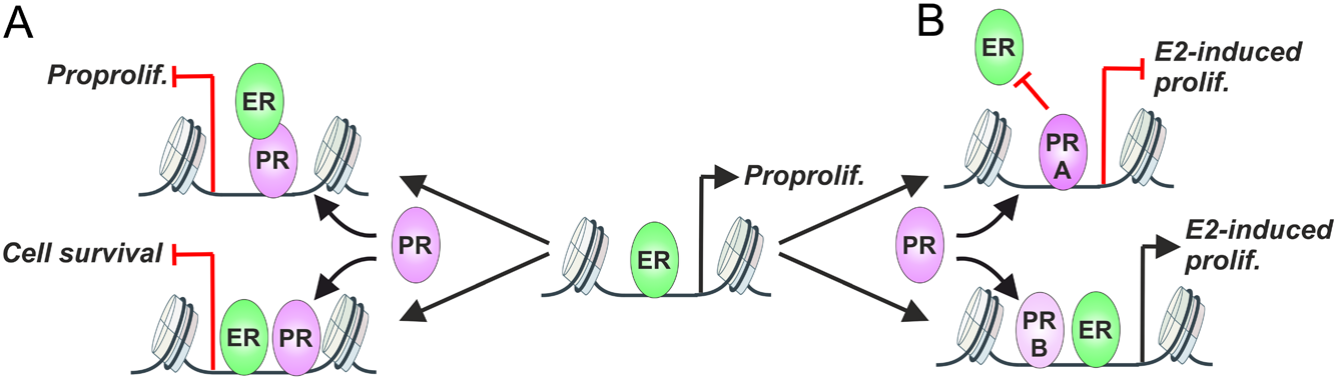
Crosstalk between PR and ER modulates BCa cell proliferation. ER regulates pro-proliferation (Proprolif) pathways in BCa. (A) Upon activation of the PR, the ER tethers to the PR (upper) or binds near or next to the PR (lower), inhibiting BCa pro-proliferation and cell survival pathways. (B) PR-A inhibits binding of the ER to chromatin and E2-induced proliferation (prolif.) (upper). PR-B induces binding of the ER, enhancing E2-induced proliferation (lower). A full color version of this figure is available at https://doi.org/10.1530/ERC-21-0038.

Recently, PR has been suggested to play a more prominent role in BCa rather than being a mere diagnostic marker (Carroll *et al.* 2017). In 2015, the Carroll laboratory reported that the activation of PR by progesterone could reprogram the chromatin occupancy of ER in a BCa cell line (Mohammed *et al.* 2015). This reprogramming resulted in thousands of new ER-binding sites not observed in BCa cells stimulated by E2 alone. Proteomic and motif analyses suggested that this had occurred through tethering of ER to chromatin-bound PR (Fig. 1A). Interestingly, the activation of PR decreased ER-driven proliferation and blocked tumor growth. This suggests that instead of being a mere marker of functional ER in BCa, the PR is a major determinant of ER-driven gene programs in BCa.

Similar, but not entirely complementary, results have been reported by the Greene laboratory (Singhal *et al.* 2016). They revealed that the PR could reprogram chromatin-binding of ER and act not only as a genomic estrogen agonist, but also as a phenotypic estrogen antagonist in ER+ PR+ BCa. However, the tethering of ER to the PR was not suggested as the main mechanism of interaction between the SRs on chromatin (Fig. 1A). Sequential (re)ChIP-seq experiments demonstrated that only some, not all, SR-bound chromatin regions harbor both ER and PR concomitantly. Even though the ER and the PR could be found on the same chromatin fragment, this does not necessarily indicate a direct interaction (i.e. tethering) between the SRs. These results suggest that the ER-PR interplay on chromatin most likely occurs through several different modes of interaction (Swinstead *et al.* 2018). Forkhead box protein A1 (FOXA1) was speculated to act as a pioneer TF, influencing the crosstalk between ER and PR (Mohammed *et al.* 2015). However, only ~50% of the PR-induced ER chromatin-binding sites contain a FOXA1 motif, and a knockdown of FOXA1 had only a minor impact on the PR-induced expression of the ER target genes (Mohammed *et al.* 2015, Singhal *et al.* 2016). It remained to be investigated whether activation of PR could alter the chromatin binding of FOXA1. Since ER has been reported to influence the chromatin occupancy of FOXA1 (Swinstead *et al.* 2016, Paakinaho *et al.* 2019*a*), it is likely that PR could act in a similar manner. As a variable number of ER-binding sites are lost after the activation of PR (Mohammed *et al.* 2015, Singhal *et al.* 2016), it is tempting to speculate that the PR could sequester FOXA1 from the ER-bound sites, thereby influencing the proliferation of BCa cells and tumor growth.

Other layers of complexity in the ER-PR interplay derive from the different isoforms of PR. The function of two alternate promoters of *PGR* give rise to two different receptors, PR-A and PR-B. The PR-A lacks 164 N-terminal amino acids of the larger PR-B isoform (Cenciarini & Proietti 2019). In other respects, the PR isoforms share the same amino acid sequence. Despite the overall similarity, while PR-A and PR-B have shared functions, they also have certain unique properties. Interestingly, in the genome-wide ER-PR crosstalk, the PR-A mainly inhibited, while the PR-B reprogrammed the chromatin-binding of ER (Singhal *et al.* 2018). This was reflected at the level of transcription. For example, estrogen-driven proliferation was attenuated by PR-A but augmented by PR-B (Fig. 1B). In theory, this kind of isoform-specific modulation of PR activity could lead to a favorable outcome of BCa. Indeed, the PR-A- and PR-B-induced gene signatures were associated with poorer and better patient survival, respectively (Singhal *et al.* 2018). Although, the above investigations strongly point to tumor suppressor capabilities of PR, more recent work has suggested that both PR-A and PR-B can exert tumo-promoting effects in ER+BCa (Truong *et al.* 2019). PR-B was revealed as a driver of BCa cell proliferation (Truong *et al.* 2019), which is complementary to the results reported by Singhal *et al.* (2018). Moreover, the Lange laboratory demonstrated that PR-A was a driver of cancer stem cell (CSC) expansion in BCa cells and that phosphorylation of PR-A was required for the expression of CSC-associated genes (Truong *et al.* 2019). Thus, the poor patient survival linked with the PR-A gene signature (Singhal *et al.* 2018), could derive from the expansion of BCa CSC. However, more investigations will be needed to fully appreciate the impact PR and its isoforms on BCa development and progression as well as the PR’s role as a potential drug target in BCa. Furthermore, it is largely unknown if and how ERβ and PR can influence each other’s transcriptional activity.

In addition to influencing the progression of BCa in cell and animal models, the status of PR correlates with patient survival (Table 1). The loss of PR expression in ER+ BCa decreases patient survival, whereas patients who have ER+ and PR+ BCa show increased survival compared to PR+ and ER- patients. Due to the evident importance of PR activity in BCa survival, clinical trials are being conducted to assess the influence of a PR agonist in the treatment of ER+ BCa (NCT03306472, NCT03024580).

**Table 1:** Influence of PGR (PR) expression on BCa patient survival.

Type	Comparison	Increased survival	*P*-value	HR	Reference
ESR1+	PGR loss vs neutral/gain	PGR neutral/gain	0.001	1.46	Mohammed *et al.* 2015
PGR-	ESR1− vs ESR1+	No difference	0.27	NA	Singhal *et al.* 2016
PGR+	ESR1− vs ESR1+	ESR1+	3.50E−02	NA	Singhal *et al.* 2016

ESR1, estrogen receptor α; HR, hazard ratio; NA, not applicable or indicated; PGR, progesterone receptor.

### Genome-wide crosstalk between the ER and the AR in BCa

Compared to our knowledge of the cross-regulatory role of PR with ER, the genome-wide interplay between AR and ER in BCa is a relatively recently recognized phenomenon. The AR is expressed in ~80% of BCa cases (McNamara *et al.* 2014), with a high expression of AR in ER+ BCa patients correlating with a better survival (Table 2). However, this is not the case in ER− BCa patients (Peters *et al.* 2009). AR interestingly regulates the same transcriptional programs in molecular apocrine BCa (AR+TNBC) cells as ER in luminal BCa cells (Robinson *et al.* 2011, McNamara *et al.* 2014). Thus, AR has been recognized as a potentially valuable drug target in TNBC, as its inhibition could offer an alternative form of therapy (McNamara *et al.* 2014, Gerratana *et al.* 2018). Moreover, the AR’s value as a drug target is strengthened by the concept that the molecular apocrine BCa and castration-resistant PCa (CRPC) cells share a core AR cistrome and target gene signature linked to cancer cell growth (Malinen *et al.* 2015). However, a recent study indicated that AR+ TNBC displayed heterogeneity in AR levels, which influenced AR-targeted therapy in combination with cell cycle inhibitors (Christenson *et al.* 2021). These results suggest that in the presence of the ER, the AR could suppress BCa cell growth, whereas the AR could promote it in the absence of ER.

**Table 2:** Influence of AR expression on BCa patient survival.

Type	Comparison	Increased survival	*P*-value	HR	Reference
ESR1+	AR low vs high	AR-high	0.002	0.22	Peters *et al.* 2009
ESR1−	AR low vs high	no difference	0.32	NA	Peters *et al.* 2009
TCGA-BRCA	AR low vs high	AR-high	1.10E-16	0.52	Ponnusamy *et al.* 2019

AR, androgen receptor; ESR1, estrogen receptor α; HR, hazard ratio; NA, not applicable or indicated; PGR, progesterone receptor; TCGA-BRCA, The Cancer Genome Atlas Breast Invasive Carcinoma.

**Figure 2:**
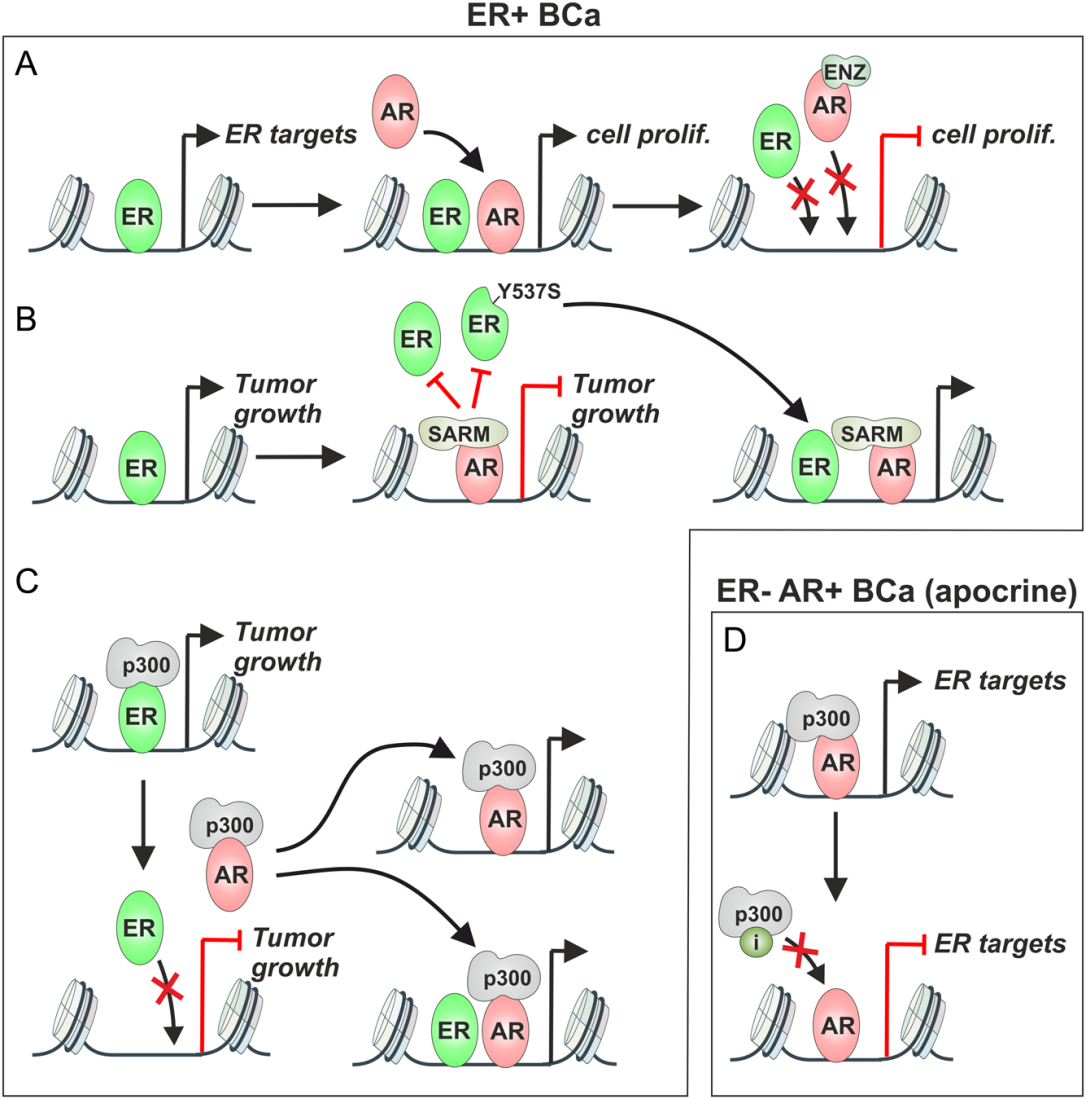
Different AR ligands modulate binding of ER to chromatin in BCa cells. (A) In ER+ BCa cells, a subset of ER binding sites (left) can be occupied by activated AR (middle), regulating cell proliferation (prolif.). ENZ inhibits the binding of AR and ER, repressing cell proliferation (right). (B) ER binding and its regulation of tumor growth pathways (left) can be inhibited by SARM-bound AR (middle). Binding of ligand-independent ER-Y537S mutant is also inhibited by AR. SARM-bound AR redistributes ER to other chromatin sites (right). (C) Agonist activated AR redistributes (squelches) EP300 from ER- to AR-binding sites. The ER is redistributed to a subset of these sites. (D) In apocrine BCa (ER-/AR+) cells, the AR can regulate the same targets as the ER in ER+ BCa cells (upper). Inhibition of EP300 by a specific acetyltransferase inhibitor (i) can repress the AR-regulated transcription (lower). A full color version of this figure is available at https://doi.org/10.1530/ERC-21-0038.

Although a high expression level of AR has been associated with increased survival of ER+ BCa patients, the Richer group found that inhibition of AR by enzalutamide (ENZ) (a second generation antiandrogen) decreased BCa cell proliferation and tumor size (D’Amato *et al.* 2016). Interestingly, these investigators showed that ER and AR could bind to the same genomic sites and that ENZ-inhibited chromatin binding not only of the AR but also the ER (Fig. 2A). These results suggest that the AR supports the chromatin-binding of ER, influencing BCa cell proliferation and tumor growth. In clinical trials, a combination of bicalutamide (first generation antiandrogen) with AI did not, however, confer any clinical benefit (NCT02910050) in ER+ and AR+ BCa patients, but a clinical trial combining fulvestrant (ER degrader) and ENZ is underway (NCT02953860).

A later study suggested that a selective AR modulator (SARM)/agonist, rather than ENZ, would be capable of inhibiting ER+ BCa tumor growth (Ponnusamy *et al.* 2019). Intriguingly, while the SARM-bound AR reduced ER occupancy on chromatin at a subset of sites, it also redistributed ER to new genomic sites (Fig. 2B). Furthermore, some of these effects were not restricted to WT ER, since AR was also able to inhibit the growth of BCa tumors expressing an estrogen-independent ER-Y537S mutant. In confirmation, a more recent investigation indicated a redistribution of ER occupancy and the inhibition of ER-induced BCa proliferation upon AR activation with an agonist (Hickey *et al.* 2021). Mechanistically, the redistribution of ER by AR was suggested to be tightly linked to a squelching of EP300 from ER- to AR-binding sites (Fig. 2C). Thus, the ER and the EP300 are redistributed from loci associated with a poor patient survival to AR-regulated loci associated with a good patient survival outcome. These results suggest that in ER+ BCa, it is activation rather than inhibition of AR that should be pursued. The differences observed between ENZ (D’Amato *et al.* 2016) and SARM/agonist (Ponnusamy *et al.* 2019, Hickey *et al.* 2021) could be attributed to the different models used in the studies. Furthermore, another study utilizing endocrine therapy-resistant BCa models revealed that the resistance (to ER inhibition) could be reversed by knockdown of AR, but not by ENZ (Chia *et al.* 2019). The apparent differences in the effect of ENZ could derive from the different concentrations of ENZ used in these studies. Since not all of the above experiments have been performed in the same cellular milieu, other TFs or coregulators (with different expression levels in the models) that influence the ER-AR crosstalk could also explain the observed differences. Since the squelching of EP300 by AR from ER-binding sites in ER+ BCa appears to be the prevalent crosstalk mechanism between the SRs (Hickey *et al.* 2021), the EP300 might act as an important regulator of AR’s action in BCa. Indeed, the activity of AR in TNBC is sensitive to the inhibition of EP300 (Garcia-Carpizo *et al.* 2019) (Fig. 2D). There are also other coregulators, such as BET family proteins, that are known to exert distinct effects on ER-regulated gene expression programs (Murakami *et al.* 2019); their presence could influence the crosstalk between AR and ER in ER+ BCa cells.

### Genome-wide crosstalk between the ER and the GR in BCa

**Figure 3:**
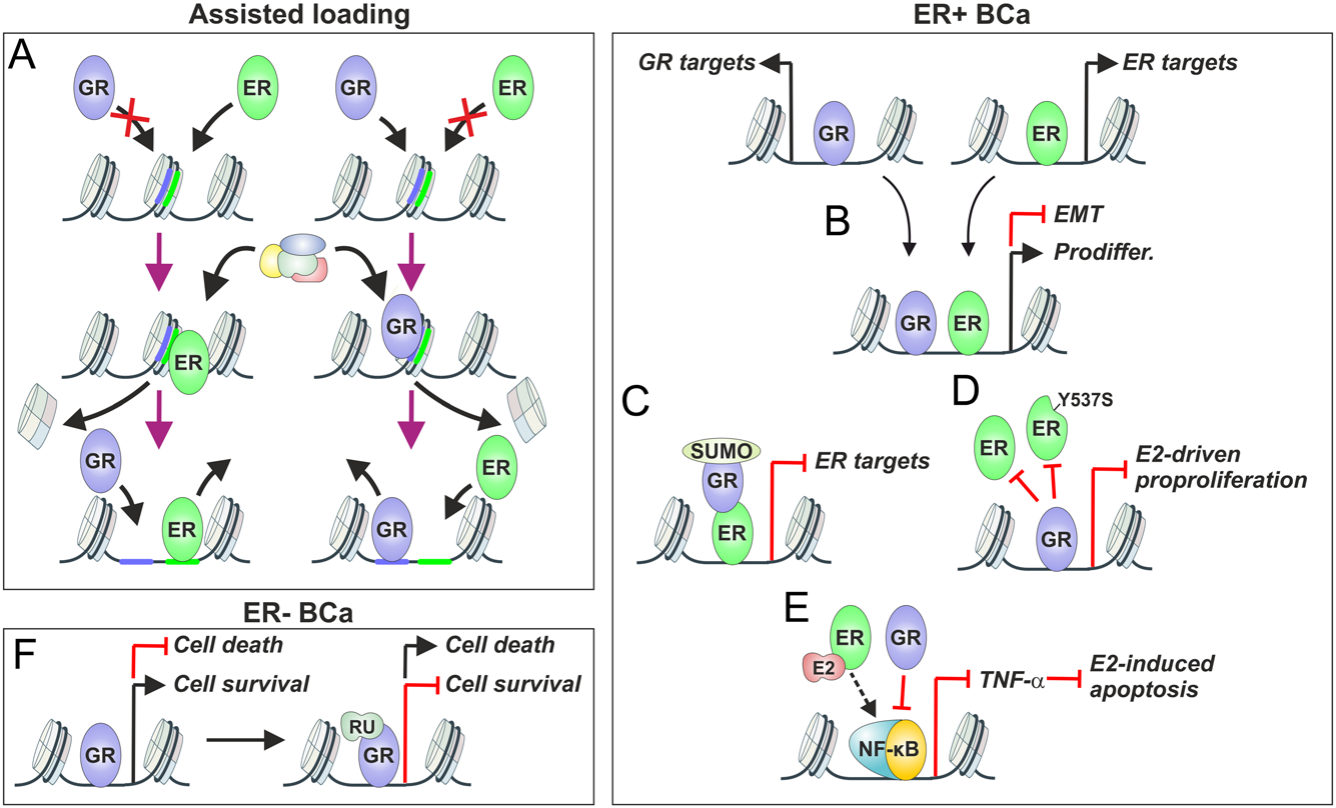
GR has multiple different mechanisms to modulate ER action in BCa cells. (A) Assisted loading model was initially shown with ER and GR. The initiating factor (ER in left, GR in right) binds to a closed chromatin region, and upon recruitment of chromatin remodeling factors, the secondary factor (GR on the left, ER on the right) can bind to the site. (B) In ER+ BCa cells, upon coactivation, GR and ER bind to the same sites, and induce pro-differentiation (Prodiffer.) and repress epithelial–mesenchymal transition (EMT). (C) SUMO-modified GR can tether to the ER and repress ER’s target genes. (D) GR can actively inhibit the chromatin-binding of ER and ligand-independent ER-Y537S mutant at regulatory sites of genes involved in E2-driven pro-proliferation. (E) In long-term E2 derived BCa cells, the GR can inhibit the action of NF-κB, thereby reducing the levels on TNF-α and E2-induced apoptosis. (F) In ER- BCa cells, GR can induce cell survival and repress cell death pathways (left). Inhibition of the GR with antagonist RU486 (RU) induces cell death and represses cell survival pathways (right). A full color version of this figure is available at https://doi.org/10.1530/ERC-21-0038.

Corticosteroids, such as dexamethasone, are widely used in the treatment of breast cancer to alleviate the side effects of chemotherapy and to treat symptoms related to advanced cancer. The genomic crosstalk in BCa cells between the ER and the GR has been the most extensively studied of the different SR pairs. Originally, the Hager laboratory demonstrated that chromatin binding of ERpBox, a DBD ER mutant that binds to GRE instead of ERE, to a subset of chromatin sites was enabled by the GR (Voss *et al.* 2011). This mechanism was termed assisted loading (Fig. 3A) and in this process, an initiator TF (such as GR) could bind to a closed chromatin site; this induced a remodeling of chromatin, thereby assisting the binding of a second TF (such as ER). The second TF was incapable of binding to the closed site without the action of the first TF. This occurred in a symmetric and enhancer-specific manner such that the dependency of TF could be reversed, that is, the ER could assist the binding of GR at some sites. It is notable that even though the TFs can bind to the same site, they do not compete for the binding site due to their rapid binding kinetics on chromatin (Paakinaho *et al.* 2017). Since the initiator TF induced remodeling of chromatin at assisted sites, chromatin remodeler complexes were postulated to play a key role in mediating assisted loading (Swinstead *et al.* 2018). The assisted loading between ER and GR has been demonstrated at a genome-wide level in mouse mammary cells (Miranda *et al.* 2013), highlighting the potential importance of the crosstalk between ER and GR in human BCa.

In human BCa patients, high levels of GR are associated with an increased survival in ER+ BCa individuals, while in ER- BCa patients, the high levels are associated with a worse survival (Pan *et al.* 2011). This relationship is seen both in pre- and post-therapy-treated patients, and the better survival of ER+ BCa patients with high GR levels occurs irrespective of PR expression level (Table 3). In ER+ BCa cells, concomitant activation of ER and GR leads to alterations in transcriptional programs that promote differentiation and decrease epithelial–mesenchymal transition (West *et al.* 2016), which is reflected as a large overlap between ER and GR on chromatin (Fig. 3B). This is reminiscent of the assisted loading reported by the Hager laboratory (Miranda *et al.* 2013). There are also other similarities, as activator protein 1 (AP-1) was shown to be an important mediator of assisted loading in mouse mammary cells (Miranda *et al.* 2013), and AP-2 motifs are enriched at many sites showing co-occupancy of ER and GR (West *et al.* 2016). Thus, it seems likely that other TFs and coregulators also participate in the crosstalk between the SRs. Whether the ER and the GR physically interact on chromatin, tethering to each other, remains an open question, although the tethering is implied by the lack of a response element for one of the receptors at the co-occupying sites (West *et al.* 2016). In support of the tethering concept and the role of post-translational modification, small ubiquitin-related modifier (SUMO)-modified GR has been reported to specifically tether to ER, resulting in the recruitment of corepressor complexes and repression of ER-driven transcription (Fig. 3C) (Yang *et al.* 2017). However, this mechanism was shown to operate only on a few selected loci, whereas at the genome- and proteome-wide level, SUMO modification of GR fine-tunes the chromatin occupancy and interactome of the receptor, impacting on gene expression in a target gene selective manner (Paakinaho *et al.* 2014, 2021). A recent study revealed that in addition to pure agonists, selective GR modulators (SGRMs) possess the ability to antagonize transcription of canonical GR target genes and thus could inhibit estrogen-induced proliferation in ER+ BCa models (Tonsing-Carter *et al.* 2019). SGRMs decreased the occupancy of the ER at several enhancers, and that the displacement of ER from chromatin by the liganded GR was associated with decreased expression of key genes mediating E2-driven proliferation (Tonsing-Carter *et al.* 2019) (Fig. 3D). The SGRM-bound GR was also able to inhibit the actions of the estrogen-independent ER-Y537S mutant. These results support the concept of developing selective SR modulators for the treatment of endocrine therapy-resistant ER+ BCa.

**Table 3:** Influence of NR3C1 (GR) expression on BCa patient survival.

Type	Comparison	Increased survival	*P*-value	HR	Reference
ESR1+ untreated	NR3C1 low vs high	NR3C1-high	0.03	0.6	Pan *et al*. 2011
ESR1+ tamoxifen	NR3C1 low vs high	NR3C1-high	7.70E−08	0.25	Pan *et al*. 2011
ESR1− untreated	NR3C1 low vs high	NR3C1-low	0.001	2.23	Pan *et al*. 2011
ESR1− chemotherapy	NR3C1 low vs high	NR3C1-low	5.80E−07	6.83	Pan *et al*. 2011
ESR1+	NR3C1 low vs high	NR3C1-high	7.80E−14	0.35	West *et al.* 2016
ESR1+ PGR-high	NR3C1 low vs high	NR3C1-high	2.30E−07	0.35	West *et al.* 2016
ESR1+ PGR-low	NR3C1 low vs high	NR3C1-high	4.10E−06	0.4	West *et al.* 2016
TNBC basal-like 1	NR3C1 low vs high	NR3C1-low	0.013	1.87	West *et al.* 2018
TNBC basal-like 2	NR3C1 low vs high	No difference	0.64	NA	West *et al.* 2018
TNBC mesenchymal	NR3C1 low vs high	NR3C1-low	0.04	1.65	West *et al.* 2018
TNBC luminal AR	NR3C1 low vs high	NR3C1-low	0.015	1.68	West *et al.* 2018

AR, androgen receptor; ESR1, estrogen receptor α; HR, hazard ratio; NA, not applicable or indicated; NR3C1, glucocorticoid receptor; PGR, progesterone receptor; TNBC, triple negative breast cancer.

As indicated earlier, high levels of GR in ER- BCa are associated with a poor patient survival (Table 3). Further analyses of patient data indicated that a similar association was evident with different subtypes of TNBC; basal-like 1, mesenchymal, and luminal AR (West *et al.* 2018). In TNBC cells, GR induces the genes related to cell survival and suppresses those related to cell death. Interestingly, this GR-mediated regulation can be reversed by using RU486 (mifepristone), a steroidal antagonist that also influences chromatin binding of the GR (Fig. 3F); in fact, RU486 is currently being tested in clinical trials for GR+ TNBC (NCT02788981). In addition to RU486, a non-steroidal SGRM, Compound A (CpdA), was similarly demonstrated to reduce GR-mediated regulation of pro-tumorigenic genes (Chen *et al.* 2015).

### Complications of the ER-GR crosstalk with other signaling pathways in BCa

The above investigations highlight the apparent overall benefit of the GR-ER crosstalk in the suppression of BCa cell growth. However, new cautionary and complicating results are emerging, not from the direct crosstalk between the SRs, but from the effects of SRs on other signaling pathways and TFs. For example, in conjunction with the ER, the proinflammatory TF nuclear factor-κB (NF-κB) regulates a subset of cell growth-related genes, resulting in a restriction and reduction of BCa cell proliferation (Franco *et al.* 2015). The GR is a well-known suppressor of NF-κB activity in immune cells (Syed *et al.* 2020). More recently, the GR has been demonstrated to suppress the actions of NF-κB also in long-term estrogen-deprived BCa cells (Fan *et al.* 2019), leading to an inhibition of TNF-α production and a complete blockade of E2-induced apoptosis and the consequent survival of cancer cells (Fig. 3E). Thus, glucocorticoids should be used cautiously in patients who have been extensive treated with AI. This caution is supported by recent observations showing that the activation of GR enhances the ability of TNBC to metastasize (Obradović *et al.* 2019). GR and ER also undergo a crosstalk with a TF signal transducer and activator of transcription 3 (STAT3) that becomes activated after phosphorylation in response to interferons, EGF, interleukin (IL-)5 and IL-6. For example, in basal-like TNBC cells, GR operates with STAT3 in a genome-wide manner to drive BCa growth (Conway *et al.* 2020). Since the activation of STAT3 reprograms binding of ER on enhancers and induces metastasis of ER+ BCa (Siersbæk *et al.* 2020), it could act as a central TF, defining the tumor-inducing or repressing role of the SR. The GR can also have an important ligand-independent activity in TNBC. The Lange laboratory has recently shown that transforming growth factor β1 (TGFβ1) could increase the phosphorylation of GR at S134 (Perez Kerkvliet *et al.* 2020), which induced the transcriptional activation of the GR in a ligand-independent manner, driving migration and anchorage-independent growth of TNBC cells. Interestingly, at least the latter effect can be inhibited by RU486.

Taken together, these results indicate that GR plays different roles in BCa cells depending on the presence of the ER as well as the activity of other signaling pathway-regulated TFs. The ER-GR crosstalk operates through several different modes in different BCa cell types and disease stages. Finally, the ER-GR crosstalk and the importance of SR expression levels for cancer survival are not restricted to BCa, as similar, though not identical, mechanisms of crosstalk have been observed between the ER and the GR in endometrial cancer (Vahrenkamp *et al.* 2018).

## Prostate cancer

PCa is the most common cancer in men and among the leading causes of cancer deaths in United States and Finland (Centers for Disease Control and Prevention 2017, Finnish Cancer Registry 2018). The primary cancer is almost always dependent on the AR signaling (Wang *et al.* 2018*a*). Nonetheless, PCa patients with localized tumor are usually initially treated with radiotherapy and/or surgery or mere active surveillance, depending on the evaluation of the risk level of the disease. For advanced disease, androgen deprivation therapy (ADT) has for a long time been the gold standard treatment (Wang *et al.* 2018*a*, Swami *et al.* 2020). Although, a recent clinical trial has indicated that chemotherapy with ADT is more efficient primary treatment than ADT alone, when there is a high metastatic burden (Sweeney *et al.* 2015). After the relapse of the primary ADT, second-line androgen deprivation or chemotherapy is typically administrated. ADT is based on either surgical or chemical castration or antiandrogens to prevent the production or action of androgens and thereby the growth of PCa cells. In antiandrogen therapy, AR action is blocked with an antagonist, such as bicalutamide, ENZ, apalutamide or darolutamide; these are compounds that compete with androgens for binding to the AR (Wang *et al.* 2018*a*, Feng & He 2019, Swami *et al.* 2020). After the initial ADT, CRPC can occur such that AR signaling is restored through variable mechanisms. This state can be treated with additional ADT. The synthesis of androgens can be blocked with abiraterone, a drug that inhibits the CYP17A1 enzyme, which catalyzes a critical step in the synthesis of androgen. Coadministration of a glucocorticoid is required to compensate for the abiraterone-induced reduction in serum cortisol and to block the compensatory increase in adrenocorticotropic hormone (ACTH). However, further resistance, such as the development of neuroendocrine PCa, which is unresponsive to further ADT, can take place (Ku *et al.* 2019, Carceles-Cordon *et al.* 2020). In addition, other SRs, such as GR, can contribute to the therapy resistance (Narayanan *et al.* 2016), emphasizing the importance of investigating how SRs interact on chromatin and together influence the development and progression of PCa. Surprisingly, there is a real scarcity of information on how ER and PR can influence AR signaling on a genome-wide scale in PCa. In the case of ER, the focus of the investigations has been on the differential role of ERα and ERβ in PCa. While early analyses suggested that ERα, but not ERβ, was expressed at various stages of PCa (Bonkhoff *et al.* 1999), more recently, both ER forms have been implicated in PCa development and tumor progression (Bonkhoff & Berges 2009). Furthermore, many PCa datasets display an increased level of ERα in more advanced cancers in comparison to less advanced cancers or benign prostate tissue (Chakravarty *et al.* 2014). On the other hand, variable levels of PR transcripts have been measured in hormone-refractory tumors (Latil *et al.* 2001). PR-B, but not PR-A, has been reported to be an independent predictor of PCa recurrence (Grindstad *et al.* 2018). Since genome-wide level information of the crosstalk between ER and PR with AR in PCa is lacking, we will focus on the interplay between AR and GR, which has been examined in PCa cells in an unbiased genome-wide fashion.

### Genome-wide crosstalk between the AR and the GR in PCa

The potential role of glucocorticoids, but not that of GR, PCa was discovered in the early 2000s, when an AR mutant from a patient was shown to be activated by glucocorticoids (Zhao *et al.* 2000, Chang *et al.* 2001). Similar promiscuous LBD mutations, L702H and T878A (L701H and T877A in early release of human genome builds), were found in PCa patients and commonly used PCa cell lines (Veldscholte *et al.* 1990, Robinson *et al.* 2015). In addition to natural and synthetic glucocorticoids (Chang *et al.* 2001), an AR with the T878A mutation was found to be activated by E2 and progesterone (Zhao *et al.* 2000), and PCa cells expressing one of the mutant ARs could obtain a growth advantage after cortisol exposure (Krishnan *et al.* 2002).

After the discovery of glucocorticoid-activated AR mutations, the GR itself was found to possess tumor suppressor activity in PCa cells (Yemelyanov *et al.* 2007). Glucocorticoids inhibited the growth of PCa cells expressing both GR and WT AR. Interestingly, the expression levels of GR were either decreased or absent in 70–85% of PCa patients compared to those with a benign form of the disease. In a follow-up study, CpdA was found to inhibit the growth of both AR- and GR-expressing PCa cells (Yemelyanov *et al.* 2008). In both studies, the restriction of cell growth was attributed to GR-mediated inhibition of MAPK signaling and decreased expression of AP-1 and NF-κB. It has been suggested that GR can repress the activity of these TFs, both indirectly by inhibiting the MAPK signaling and directly through CpdA-induced transrepression. Interestingly, GRβ, an alternative splicing isoform of GR that does not bind glucocorticoids (Timmermans *et al.* 2019) possessed the ability to modify the growth of PCa cells, as its depletion decreased PCa cell proliferation (Ligr *et al.* 2012), suggesting that the GRβ could modulate the tumor suppressor capability of the full-length GR in PCa.

The initial crosstalk between AR and GR in PCa was observed in their similar response to chromatin binding after FOXA1 depletion from PCa cells (Sahu *et al.* 2011). Depletion of FOXA1 resulted in a reprogramming of the AR and GR chromatin occupancy; some binding sites were unchanged or lost and more sites were gained. These gained and lost chromatin-binding sites were also reflected in the capability of the SRs to regulate transcription, indicating that the AR and the GR behave similarly with the FOXA1 on chromatin in PCa cells. Indeed, there is a large overlap between the SR-binding sites in PCa cells, and FOXA1 has been postulated to specify unique binding sites of AR and GR, depending on the PCa cell line (Sahu *et al.* 2013). Conversely, activation of AR can redistribute FOXA1 on chromatin (Paakinaho *et al.* 2019*a*). The importance of FOXA1 for PCa biology is also supported by the findings that the expression level and mutations of FOXA1 substantially influence PCa progression (Sahu *et al.* 2011, Adams *et al.* 2019, Parolia *et al.* 2019).

**Figure 4:**
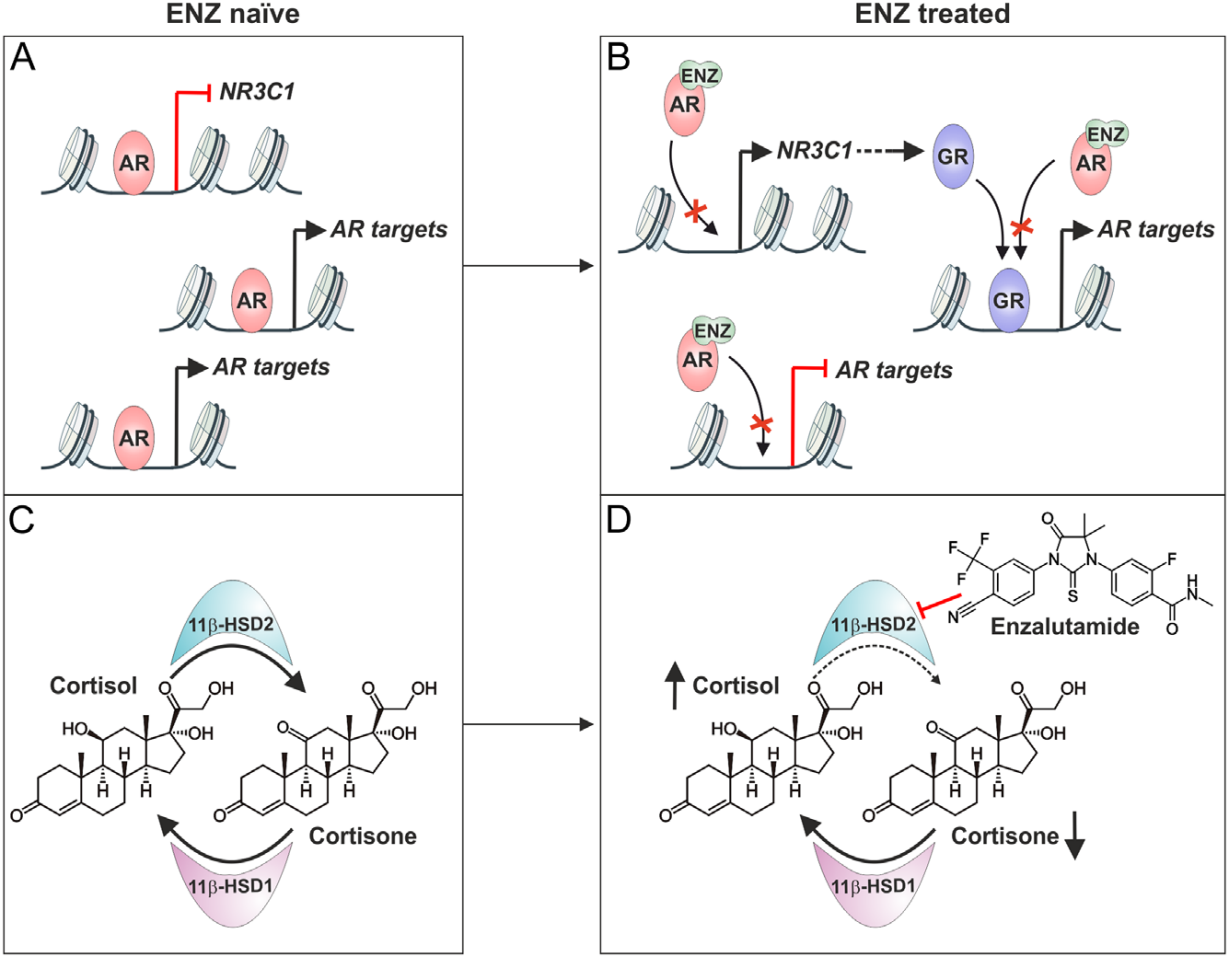
GR can replace ENZ-inhibited AR in PCa. (A) In therapy naïve PCa cells, the AR actively represses the transcription of the GR gene (*NR3C1*). (B) In ENZ treated PCa cells, the activity of the AR is inhibited, leading to a de-repression of *NR3C1*. GR can substitute for the ENZ-inhibited AR at some but not at all of the AR’s binding sites. (C) Active glucocorticoid, cortisol, is converted by 11β-HSD2 to its inactive metabolite cortisone, and cortisone is converted back to cortisol by 11β-HSD1. (D) In ENZ-treated PCa cells, the protein levels of 11β-HSD2 are decreased, leading to elevated levels of cortisol and reduced levels of cortisone. A full color version of this figure is available at https://doi.org/10.1530/ERC-21-0038.

A major breakthrough in clarifying the crosstalk between the AR and the GR came from the Sawyers group (Arora *et al.* 2013). These investigators discovered that resistance to antiandrogens in PCa could occur through the replacement of AR by the GR. They postulated that the AR represses the GR gene (*NR3C1*) (Fig. 4A), which is alleviated upon long-term ENZ treatment, resulting in an enhanced expression of GR and a substitution of the AR by the GR in transcriptional regulation (Fig. 4B). Interestingly this replacement occurs at only around half, not all, of the AR-bound chromatin sites. Furthermore, the FOXA1 motif is enriched at the GR-replaced AR-binding sites, suggesting that it plays a role in the GR-mediated resistance to ENZ in PCa cells. In support of this concept, inhibition of GR was found to rescue ENZ sensitivity to prevent tumor growth (Arora *et al.* 2013), and furthermore, a depletion of GR significantly decreased the initiation and progression of resistant PCa tumors (Isikbay *et al.* 2014). One particular GR target gene, serum and glucocorticoid-regulated kinase 1 (SGK1) has been shown to have a prominent role in ENZ resistance (Isikbay *et al.* 2014); inhibition of SGK1 decreased PCa cell viability, while its overexpression increased tumor initiation. Thus, GR and its target gene products play an important role in antiandrogen resistance.

Subsequently, the importance of GR has been confirmed in PCa patient material. While the levels of GR initially rise in PCa during ADT, the levels can decrease to pre-castration levels due to restored AR signaling in castration-resistant PCa (Xie *et al.* 2015). In primary PCa, the expression of GR is reduced, but it is restored in PCa metastases, with ENZ-treated patients showing a higher GR expression than therapy naïve patients (Shah *et al.* 2017, Puhr *et al.* 2018). These results are thus complementary to data from preclinical models, showing significantly increased levels of GR upon long-term abiraterone or ENZ treatment (Arora *et al.* 2013, Puhr *et al.* 2018). As an outcome of GR expression, patients with a high expression of GR have a poor outcome to ENZ treatment, and high levels of GR are associated with a poor survival (Arora *et al.* 2013, Puhr *et al.* 2018). Regardless of the expression levels, the GR is postulated to be a crucial player in both antiandrogen resistant and therapy naïve PCa (Puhr *et al.* 2018). In addition to the GR, the Sharifi laboratory has indicated that glucocorticoid metabolism plays an additional key role in the maintenance of ENZ resistance (Li *et al.* 2017). The formation of the bioactive glucocorticoid, cortisol, is regulated by the 11β-hydroxysteroid dehydrogenase 1 (11β-HSD1) and 2 (11β-HSD2) (Timmermans *et al.* 2019). The 11β-HSD2 enzyme converts cortisol into the biologically inactive cortisone that can be converted back to cortisol by the 11β-HSD1 (Fig. 4C). Interestingly, PCa cell models and patients treated with ENZ display decreased levels of 11β-HSD2 through autocrine motility factor receptor (AMFR) ubiquitin E3 ligase-mediated degradation (Li *et al.* 2017). The decrease in 11β-HSD2 elevates the level of cortisol (Fig. 4D), which together with the increased amount of GR, leads to a systemic activation of glucocorticoid signaling and antiandrogen resistance. Furthermore, this is not restricted to tumor tissue, as patients treated with ENZ show a systemic rise in cortisol levels (Alyamani *et al.* 2020).

As indicated above, the AR signaling is an important regulator of GR expression in the prostate. Indeed, most PCa patients and PCa cell lines display high levels of either AR or GR. This suggests that there is an inverse correlation in the expression between the two SRs (Xie *et al.* 2015). This inverse correlation can be explained by a direct repression of the GR gene (*NR3C1*) by the AR, via an intronic prostate-specific enhancer in the *NR3C1* (Arora *et al.* 2013). Subsequent studies have revealed that the enhancer is repressed through an AR-induced and EZH2-mediated mechanism that is lost in ENZ-resistant PCa cells (Shah *et al.* 2017). The repression can be restored by a BET family inhibitor JQ1. In addition, GATA-binding factor 2 (GATA2), mediator complex (Yuan *et al.* 2019) and corepressor transducin-like enhancer protein 3 (TLE3) (Palit *et al.* 2019) have been shown to influence the AR-mediated repression of *NR3C1*. The functionality of the enhancer was proven by its CRISPR-Cas9-mediated mutation (Shah *et al.* 2017, Yuan *et al.* 2019).

**Figure 5:**
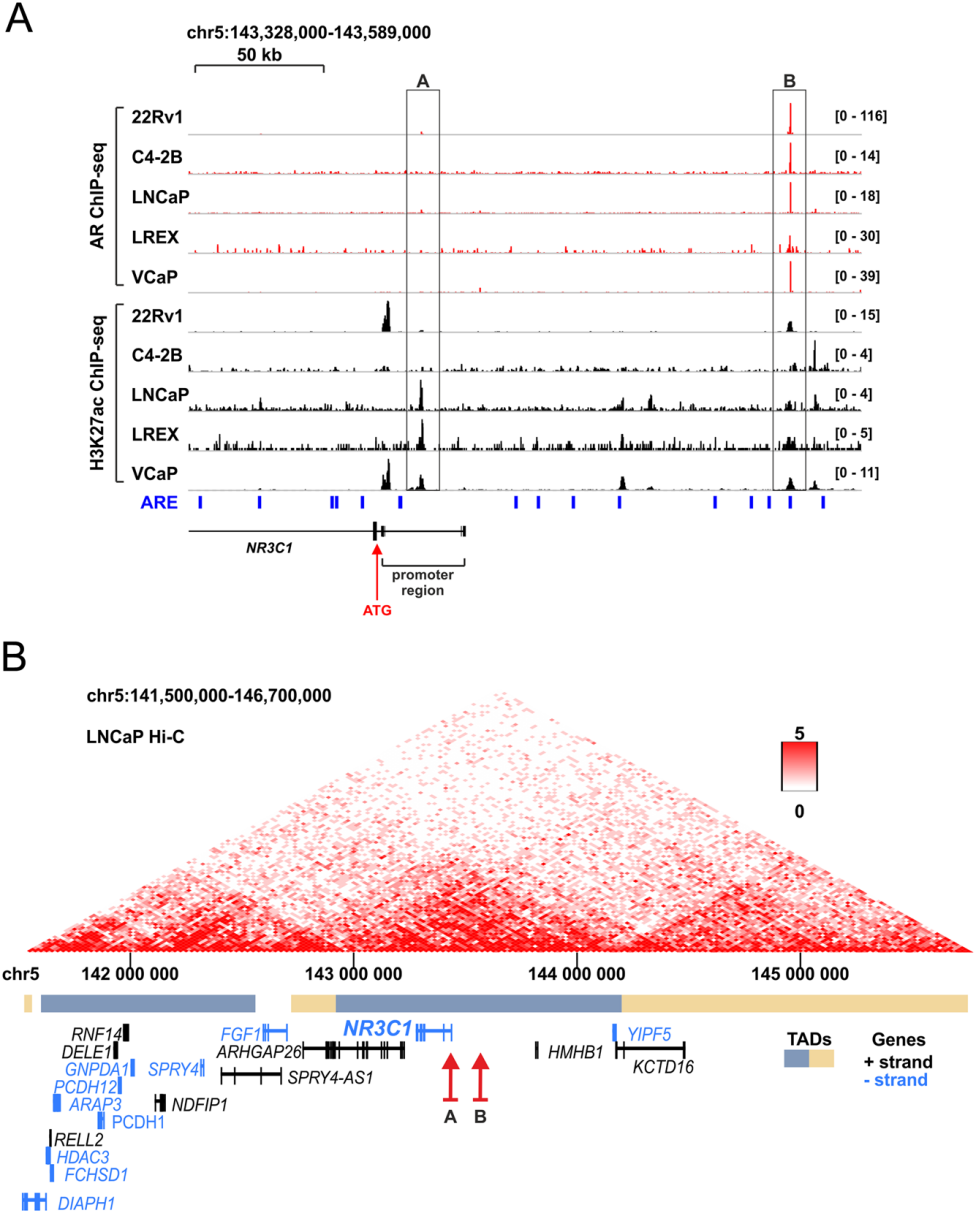
Localization of the AR-regulated genomic region that mediates the repression of *NR3C1*. (A) Genome browser tracks of AR ChIP-seq from 22Rv1 (GSE94013), C4-2B (GSE40050), LNCaP (GSE40050), LREX (GSE51497), and VCaP (GSE56086) cells (red color). Genome browser tracks of H3K27ac ChIP-seq from 22Rv1 (ENCSR391NPE), C4-2B (ENCSR279KIX), LNCaP (GSE118514), LREX (GSE103449), and VCaP (ENCSR597ULV) cells (black color). Data are mapped to human hg38 genome. Location of ARE motifs (based on HOMER are.motif sequence) shown as blue bars below the tracks. The location of *NR3C1* promoter region is depicted as well as the initiation codon ATG (red arrow). Enhancer A: AR-regulated enhancer region as suggested in Shah *et al.* (2017) and Yuan *et al.* (2019). Enhancer B: AR-regulated enhancer region as suggested in Xie *et al.* (2015). (B) *NR3C1* and both suggested AR-regulated enhancers reside within the same TAD (topologically associating domain). The AR-regulated enhancer region proposed by Shah *et al.* (2017) and Yuan *et al.* (2019) (enhancer A) and suggested by Xie *et al.* (2015) (enhancer B) (red arrows) are within the same TAD based on Hi-C data from LNCaP cells (ENCSR346DCU). Data were obtained using 3D Genome Browser (Wang *et al.* 2018*b*). The interaction matrix shown above with a scale from 0 to 5. Genes on the positive strand are shown in black, and genes on the negative strand are shown in light blue. TADs are distinguished with different colors. A full color version of this figure is available at https://doi.org/10.1530/ERC-21-0038.

The 'intronic enhancer' (Arora *et al.* 2013) that lacks AREs and only weakly binds AR is surprisingly located in the promoter region of *NR3C1,* very close to the initiation codon ATG (Fig. 5A) (Timmermans *et al.* 2019). In contrast, another study suggested that the enhancer-mediating the repressive effect of AR on the expression of *NR3C1* would be located far more upstream from the gene promoter (Xie *et al.* 2015). This upstream enhancer contains an ARE sequence and shows prominent binding of AR (Fig. 5A). Thus, the decrease of GR levels after CRISPR-Cas9-facilitated deletion of the putative 'intronic enhancer' (Shah *et al.* 2017, Yuan *et al.* 2019) may be due to the disruption of the promoter function rather than that of its interaction with the AR with it. However, it remains to be investigated how disruption of the upstream enhancer would influence the expression of* NR3C1*. Since both putative enhancers reside within the same topological associating domain (Fig. 5B) (ENCODE Project Consortium 2012, Wang *et al.* 2018*b*), the upstream enhancer could, in fact, loop to the promoter.

In addition to GR, AR splice variants are well-known drivers of antiandrogen resistance in PCa (Blatt *et al.* 2021). The splice variants, such as AR-V7, lack the LBD and are therefore constitutively active and do not respond to ENZ treatment. Interestingly, ENZ- and abiraterone-resistant PCa patients show a negative correlation between AR-V7 and GR expression (Shah *et al.* 2017). Thus, AR-V7 and the replacement of the AR by the GR might represent a mutually exclusive mechanism of antiandrogen resistance. However, both AR-V7 and GR display high expression levels in some individual patients (Shah *et al.* 2017). This suggests that antiandrogen resistance could be derived simultaneously or sequentially from both AR-V7 and GR. Nevertheless, larger patient cohorts will need to be analyzed to estimate the relative contribution of AR-V7 vs that of GR to the antiandrogen resistance. Compared to PCa, the occurrence and the role of ER splice variants are relatively unknown in therapy naïve or endocrine-resistant BCa (Blatt *et al.* 2021). However, ER gene fusions with other proteins, such as YAP1, have been observed to occur in BCa. These types of SR fusions have not been demonstrated for the AR in PCa. However, the ER gene fusions do not seem to be involved in the crosstalk between SRs in BCa.

Overall, the above data indicate that glucocorticoids should be used with caution, especially in patients undergoing ENZ therapy. Synthetic glucocorticoids, such as prednisolone, are widely used to alleviate therapy-related side effects, to reduce inflammation and to counteract the decrease in cortisol levels due to abiraterone treatment (Narayanan *et al.* 2016). Thus, alternative ways of modulating GR activity in PCa have been under investigation. Interestingly, an SGRM has been reported to decrease the GR-mediated PCa cell proliferation and CRPC tumor growth and viability without inhibiting the activity of the AR (Kach *et al.* 2017). Therefore, two different SGRMs in combination with ENZ are currently being evaluated in clinical trials to treat metastatic PCa (NCT03437941, NCT03674814). Even though RU486 can bind to AR and PR in addition to GR (Kach *et al.* 2017), a combination therapy with RU486 and ENZ is under evaluation in clinical trials in hormone-resistant PCa (NCT02012296). Based on the results from the preclinical models, targeting of the DNA binding half-site sequence of ARE/GRE by a pyrrole-imidazole polyamide instead of the GR may emerge as an alternative approach to block the actions of both the AR and GR (Kurmis *et al.* 2017). Taken together, these results support the importance of augmented GR signaling as a key player in antiandrogen resistance of PCa, warranting further research on developing novel approaches to target the signaling in PCa tissue without compromising the beneficial effects of glucocorticoids at a system-wide level.

## Differential role of the GR in the steroid receptor crosstalk in breast and prostate cancers

The AR and the ER play analogous roles in PCa and BCa, respectively. Both SRs are frequently mutated or alternatively spliced in response to endocrine therapy (suppression of hormone availability or receptor activity), resulting in a resistant disease (Metcalfe *et al.* 2018). Moreover, the activity of both receptors can be similarly reprogrammed during the progression of these cancers. For example, the chromatin-binding of the AR and ER can be pioneered by FOXA1 (Hurtado *et al.* 2011, Sahu *et al.* 2011), mutations of FOXA1 impact on the activity of both the AR and the ER (Adams *et al.* 2019, Parolia *et al.* 2019, Arruabarrena-Aristorena *et al.* 2020), and both SRs also affect the chromatin binding of FOXA1 (Paakinaho *et al.* 2019*a*). However, the characteristics of the GR-ER crosstalk in BCa and those of the GR-AR crosstalk in PCa differ from each other. In BCa cell models, it is well established that the activation of GR can expand the binding of ER to chromatin and vice versa (Miranda *et al.* 2013, West *et al.* 2016), mainly through an assisted loading mechanism (Swinstead *et al.* 2018). This has not been explicitly demonstrated for the GR and the AR in PCa. The AR and the GR share binding sites in PCa cells, and at some chromatin sites, GR binding is enhanced upon activation of both SRs, which is reminiscent of assisted loading (Sahu *et al.* 2013). However, AR binding seemed unchanged upon GR activation. Thus, while the crosstalk between the GR and the ER in BCa is symmetric, that between the GR and the AR in PCa appears to be asymmetric. Even though coactivation of ER and GR in BCa leads mainly to a synergistic transcription and expression of ER target genes (West *et al.* 2016), the expression of some ER target genes was restricted upon activation of GR. In contrast, PCa cell models showed no clear synergy between the GR and the AR in target gene regulation (Sahu *et al.* 2013). At some selected target genes, it seemed that the expression of AR-regulated genes was restricted by GR, while that of GR-regulated genes was conversely enhanced by AR. Some of the differences between the GR-ER and GR-AR crosstalk may, however, derive from cell models: MCF-7 cells that endogenously express both ER and GR were used as the model for BCa experiments (West *et al.* 2016), but most PCa experiments were performed in LNCaP-1F5 cells expressing endogenous AR and rat GR from an engineered, integrated gene (Sahu *et al.* 2013). Thus, it is currently unclear how the crosstalk between GR and AR in PCa occurs in a more natural setting of PCa cells. Since the expression of AR and that of GR are usually inversely correlated, there are only a few native PCa cells, such as VCaP and CWR22RV1 cells, that endogenously express both GR and AR (Puhr *et al.* 2018). For example, VCaP cells were used to show that GR and AR chromatin-binding sites overlap (Sahu *et al.* 2013), but it remains to be determined how the SRs influence each other’s chromatin binding.

Furthermore, in the case of the crosstalk linked to gene repression, the AR and the ER operate differentially with the GR. In BCa, the activation of both GR and ER can lead to a redistribution of ER from some sites to new sites that cannot be occupied by ER without GR activation (Tonsing-Carter *et al.* 2019). Nonetheless, it is not known how or even if ER represses GR activity in BCa. However, the repression of GR-regulated genes by ER has been observed (Miranda *et al.* 2013, West *et al.* 2016, Yang *et al.* 2017). Thus, it does seem that at least GR can actively repress the activity of ER. As indicated earlier, AR does not repress the activity of GR, but the expression of *NR3C1* was blocked in PCa models (Shah *et al.* 2017). Moreover, the expression of GR is enhanced upon inhibition of AR by ENZ, leading to the replacement of the AR in transcriptional regulation (Arora *et al.* 2013). A similar mechanism of therapy resistance where the GR substitutes for the ER in transcriptional regulation has not been observed in endocrine-resistant BCa. As indicated earlier, no mechanism has been elucidated for GR-mediated suppression of AR target genes (Sahu *et al.* 2013). The suppression could be due to GR-mediated repression of TFs, such as AP-1 and ETS factors (Yemelyanov *et al.* 2007), especially since the latter factors are known as collaborating TFs of the AR in PCa (Yu *et al.* 2010, Zhang *et al.* 2019). Thus, the GR could indirectly restrict the action of AR.

Finally, and interestingly, the activity of GR is rather similar in TNBC models and ENZ-resistant PCa models. where the GR drives the progression of the disease (Arora *et al.* 2013, West *et al.* 2018). Since the GR acts similarly in BCa and PCa in the absence of ER or AR, respectively, this is convincing evidence that the differential crosstalk of GR with the AR and the ER stems from the inherent activity of the ER and AR within the cells. Thus, while the activity of GR is similar in BCa and PCa cells, the crosstalk of GR with ER and AR differs as a result of their differential chromatin binding and recruitment of interaction partners (TFs and coregulators). Indeed, even though FOXA1 plays a similar role with the SRs, AR has a more pronounced effect on the chromatin binding of FOXA1 in PCa cells than ER in BCa cells (Paakinaho *et al.* 2019*a*). Moreover, depletion of FOXA1 generally decreases the binding of ER to chromatin in BCa cells (Hurtado *et al.* 2011), but in PCa cells, the occupancy of the majority of AR-binding sites remains unchanged and new binding sites are generated (Sahu *et al.* 2011). This highlights the importance of investigating how other TFs and coregulators impact on the crosstalk between different SRs.

As indicated earlier, glucocorticoids are widely used in the treatment of PCa and BCa patients, for example, to alleviate the unwanted effects of chemotherapy and to reduce inflammation. Even though GR has tumor-promoting effects in TNBC and in ENZ-resistant PCa, the overall clinical benefit of glucocorticoids outweighs their disadvantages. Thus, a more viable option to treat TNBC or GR-induced ENZ-resistant PCa would be to inhibit the action of the GR through other signaling pathways, while retaining the beneficial systemic effect of glucocorticoids. In ENZ-resistant PCa, these kinds of pathways could be BET (Shah *et al.* 2017) or PI3K/AKT (Adelaiye-Ogala et al. 2020), whereas in TNBC, STAT3 (Conway *et al.* 2020) or p38 MAPK (Perez Kerkvliet *et al.* 2020) could be targeted. Future investigations will determine if sufficient attenuation of GR signaling can be obtained through these pathways or if other yet to be discovered pathways will be more effective.

## Future perspectives

SRs are important transcriptional regulators in many different cancers (Dhiman *et al.* 2018), and the revelation of the crosstalk between the SRs opens a new avenue of targeting these diseases (De Bosscher *et al.* 2020). Overall, we propose that the physiological effects of steroid hormones in tissues are dictated not by cognate hormone-SR pairs but instead by the crosstalk between SRs and their interaction with different transcriptional complexes. All transcriptional programs in steroid target tissues and steroid-dependent diseases are likely to be governed by the crosstalk between the SRs. This crosstalk is naturally dependent on the concentrations and types of ligands present at any given time. The development of novel, more targeted therapies with fewer side effects will have to be based on a better understanding of the molecular mechanisms of this crosstalk. The crosstalk between different pairs of SRs is thus an important aspect that should be considered in the treatment of steroid hormone-regulated cancers. In particular, the crosstalk of the GR with the ER in BCa and that with the AR in PCa will be particularly critical for the development of therapies in the future (Kach *et al.* 2015). Since these SRs can reprogram each other’s chromatin binding (Miranda *et al.* 2013) as well as binding of other cancer-relevant TFs, such as FOXA1 (Swinstead *et al.* 2016, Paakinaho *et al.* 2019*a*), the regulation of the chromatin landscape of hormone-dependent cancers warrants a thorough scrutiny in future investigations. Many of the chromatin remodeling complexes have become mutated, deleted, or dysregulated in cancers (Valencia & Kadoch 2019), including BCa (Nagarajan *et al.* 2020) and PCa (Sandoval *et al.* 2018), potentially influencing the chromatin binding of SRs and the capability of SRs to reprogram the chromatin occupancy of other TFs. Indeed, the loss of the SWI/SNF subunit ARID1A influences ER activity in BCa by altering the BET family activity at the receptor-bound enhancers (Nagarajan *et al.* 2020). Furthermore, the loss of CHD1 contributes to the antiandrogen resistance in PCa, including GR-driven processes (Zhang *et al.* 2020). Thus, improved knowledge of the remodelers and the impact of their dysregulation on SR crosstalk should yield important information on how to improve targeting of the SRs in BCa and PCa.

One aspect that has been rather neglected in the SR crosstalk is the effect of steroid hormone abundance and the contribution of precursor hormones to the crosstalk. Many, if not all, investigations into SR crosstalk have been performed with saturating hormone concentrations. It is not known how physiological levels of steroid hormones and overall steroid hormone exposure influence the SR crosstalk. Studies addressing these questions are crucially important, since also the metabolism of steroid hormones is altered in BCa and PCa (Capper *et al.* 2016). Treatment of PCa tumors with ADT or abiraterone leads to reduced levels of androgens (Knuuttila *et al.* 2019), although in CRPC, the malignancy can maintain its growth by intratumoral production of androgens (Montgomery *et al.* 2008, Narayanan *et al.* 2016). The inhibition of steroid hormone metabolism can also lead to elevated levels of adrenal androgen precursors, which in turn may activate promiscuously mutated AR (Chang *et al.* 2001). Furthermore, many components of the steroidogenesis pathway are dysregulated in PCa, leading to a sustained production of androgens despite ADT (Narayanan *et al.* 2016). As well as the tumor, the PCa stroma can increase its androgen metabolism through TGFβ1 produced in the tumor’s microenvironment (Piao *et al.* 2013). Interestingly, while the expression of GR is reduced in PCa as compared to benign tissue, the PCa stroma appears to maintain its GR expression levels (Mohler *et al.* 1996). Thus, the GR could have wider effects on PCa through the stroma. The changes in steroid hormone metabolism in BCa are less well-known. The occurrence of different steroids in breast cancer has been reviewed (Africander & Storbeck 2018), but little is known about the changes occurring in steroid hormone metabolism after treatment with ER antagonists or AI. The abundance of steroid hormones is likely to affect SR crosstalk (Reddy *et al.* 2012). Furthermore, many steroid hormones are secreted in cycles, for example, an ultradian rhythm for cortisol and the menstrual cycle for estrogen and progesterone. Thus, the hormone concentrations may have a varying effect, depending on the moment of time and therapies used for cancer treatments.

In addition to chromatin, the interactomes of SRs could help to find novel mechanisms and subsequent drug targets influencing the crosstalk. Several chromatin proteomics methods have been developed, including ChIP-SICAP (Rafiee *et al.* 2016) and qPLEX-RIME (Papachristou *et al.* 2018). These methods have not yet been utilized in studies addressing the SR crosstalk, although the GR and the AR have been shown to possess a highly overlapping set of interacting chromatin proteins (Lempiäinen *et al.* 2017). Furthermore, an agonist-induced post-translational modification has been shown to regulate the interaction of GR with coregulators on chromatin (Paakinaho *et al.* 2021). Finally, oligomerization of the receptor should also be considered as a potential step in the regulation of the SR crosstalk. Many SRs form higher structures than dimer oligomers upon DNA binding (Presman *et al.* 2016). In the case of GR, it has been demonstrated the receptor’s transcriptional activity is increased if the receptor is in a higher oligomerization state prior to chromatin binding (Paakinaho *et al.* 2019*b*). It was also postulated that SRs could form atypical oligomers with each other (Jiménez-Panizo *et al.* 2019, De Bosscher *et al.* 2020), which would indeed influence the SR crosstalk. Since oligomerization of TFs has been thought to activate EP300 (Ortega *et al.* 2018) and inhibition of EP300 can restrain the crosstalk and assisted loading between non-SR TFs (Goldstein *et al.* 2017), receptor oligomerization to an atypical or higher state, via EP300 could potentially influence the crosstalk of SRs. The role of coregulators, such as EP300, could be investigated with the proteomic approaches described above.

The genome-wide crosstalk between non-SR TFs is thought to operate like the crosstalk between SRs. For example, the crosstalk between STAT3 and NF-κB during the acute phase response in hepatocytes (Goldstein *et al.* 2017) resembles that between ER and GR in BCa cells (Miranda *et al.* 2013), that is, the crosstalk occurs at some but not all TF-binding sites. The activity of SR in BCa and PCa can be modulated via other signal-activated TFs, such as STAT3 and NF-κB. The activation of STAT3 in BCa cells expands the chromatin-binding of ER, driving the metastasis of the disease (Siersbæk *et al.* 2020). Similarly, the crosstalk between GR and STAT3 can impact on cell growth of basal-like TNBC (Conway *et al.* 2020). In the case of NF-κB, the chromatin-binding of ER in BCa (Franco *et al.* 2015) and that of AR in PCa cells (Malinen *et al.* 2017) are expanded by TNF-α. In both cancers, NF-κB most likely influences the outcome of the disease. Since in addition to coregulators, ChIP-SICAP (Rafiee *et al.* 2016) and qPLEX-RIME (Papachristou *et al.* 2018) can capture the TFs interacting with the SRs, currently unknown partners mediating the crosstalk could be discovered in the future by exploiting proteomic techniques.

As indicated earlier, for some of the SRs, there is little to no genome-wide information on how they influence cancer progression and development or how they influence each other’s chromatin binding. Whether estrogen and progesterone signaling can influence PCa cells and how the ER and PRs interact with the AR are also rather underexplored areas. The MR has been the least extensively studied SR in BCa and PCa. However, some investigators have explored the MR’s potential role in both cancers. Both glucocorticoids and mineralocorticoids, together with PR, can decrease BCa cell proliferation (Leo *et al.* 2004), and a high cytoplasmic expression of MR has been associated with a poor survival of ER+/PR+/HER2- BCa patients (Jääskeläinen *et al.* 2019). In PCa, abiraterone treatment can result in excess of aldosterone and its precursors, which increases the risk for hypertension and cardiovascular diseases (Pia *et al.* 2013, Narayanan *et al.* 2016). This can be bypassed by administering MR antagonists or suppressing ACTH production with glucocorticoids. Interestingly, the MR has been claimed to have a potential role in ENZ resistance in PCa (Shiota *et al.* 2018).

In conclusion, there are many aspects of SR crosstalk that still need to be resolved. For example, only the influence of the relatively unknown SRs, but also how oligomerization, the chromatin landscape, and other TFs and coregulators influence the crosstalk between the receptors are relatively unexplored areas. In many cases, a combined therapy to target multiple pathways and factors could well be beneficial in overcoming therapeutic resistance in these hormonal cancers (Boumahdi & de Sauvage 2020, Carceles-Cordon *et al.* 2020). It should be emphasized that the mechanical aspects of SR crosstalk indicated above need to be supported by data from clinical samples. Indeed, there are several ongoing clinical trials testing the importance of the SR crosstalk in patients (NCT03306472, NCT03024580, NCT02953860, NCT02788981, NCT03437941, NCT03674814, NCT02012296), and TF interactomes have been analyzed from patient samples (Siersbæk *et al.* 2020). Moreover, single cell transcriptomics (scRNA-seq), chromatin accessibility (scATAC-seq) and spatial transcriptomic analyses will soon be able to reveal the level of cellular heterogeneity in the crosstalk, initially in preclinical models and subsequently in clinical cancer samples.

## Declaration of interest

The authors declare that there is no conflict of interest that could be perceived as prejudicing the impartiality of this review.

## Funding

V P and J J P were supported by the Academy of Finland http://dx.doi.org/10.13039/501100002341, the Cancer Foundation Finland, and the Sigrid Jusélius Foundation.

## Author contribution statement

V P and J J P wrote the paper.
